# Canadian Professional Association Resources on Diet and Tooth Decay: Website Content Analysis

**DOI:** 10.2196/59474

**Published:** 2026-01-20

**Authors:** Meg Fotheringham, Laura E Forbes, Petros Papagerakis, Jessica Lieffers

**Affiliations:** 1 College of Pharmacy and Nutrition, University of Saskatchewan, 107 Wiggins Road, Saskatoon, SK, S7N5E5, Canada, 1 306-966-8594; 2 Department of Family Relations & Applied Nutrition, University of Guelph, Guelph, ON, Canada; 3 Faculty of Dental Medicine, Laval University, Laval, QC, Canada

**Keywords:** diet, nutrition, tooth decay, internet, professional association, content analysis

## Abstract

**Background:**

Tooth decay is an important public health concern affecting individuals of all ages. Dietary intake is critical to tooth decay prevention as both the types of foods and beverages consumed and how food and beverages are consumed (eg, frequency) can impact risk. Foods and beverages can both protect against and promote tooth decay.

**Objective:**

This study aims to explore information targeted to the public on diet and tooth decay available from websites of professional organizations and regulatory bodies in Canada and the readability of this information.

**Methods:**

Canadian websites of regulatory bodies and professional organizations for dietitians, oral health professionals, nurses, and physicians in Canada were thoroughly searched by 2 researchers (MF and JL) from June to August 2020 for information related to diet and tooth decay targeted to the public. Web pages were downloaded and underwent content analysis using NVivo software (version 12; QSR International). For each website, approximately 2 web pages focused on diet and tooth decay were selected to undergo readability testing.

**Results:**

Overall, 213 web pages from 23 websites were found to contain information on diet and tooth decay. Of the 23 websites analyzed, 12 (52%) were from dental organizations, 7 (30%) from dental hygiene organizations, and only 1 (4%) from a dietitian organization. Messaging was present on numerous web pages focusing on diet and tooth decay and those that have other focuses (eg, oral hygiene tips for infants and children and general oral health tips). Messaging depth varied across all web pages, ranging from general advice (eg, consume a “healthy diet”) to specific recommendations (eg, amount of juice to consume per day). Many web pages targeted parents and school-aged children; fewer web pages targeted other age groups (eg, adolescents and older adults). Three major themes were identified: Foods, Beverages, and Behaviors to Limit; Foods, Beverages, and Behaviors to Choose; and Mixed and Other Unclear Messages. The most frequently discussed topic was sugar (mentioned in 67% of web pages). Sugar was often discussed in the context of limitation, infant feeding, and strategies for tooth-friendly consumption. The average Flesch-Kincaid grade level was 7.1 (SD 1.8), which exceeds the grade 6–level recommendation.

**Conclusions:**

This analysis of web pages found that diet and tooth decay was heavily discussed throughout websites for Canadian health professional organizations, with many web pages targeting parents and school-aged children. The readability of many analyzed web pages was above the recommended grade 6 reading level. The development of comprehensive national guidelines related to diet and tooth decay would be helpful to ensure that consistent and clear messaging is available on this topic.

## Introduction

Oral diseases (eg, tooth decay, periodontal disease, edentulism, and oral cancer) are the most prevalent chronic diseases across the globe affecting 3.5 billion people [1]. More specifically, untreated tooth decay is the most common chronic disease worldwide with 2 billion people having untreated decay in their permanent teeth and 514 million children having untreated decay in their deciduous teeth [1,2]. In Canada, tooth decay is also a public health concern. The 2007-2009 Canadian Health Measures Survey, which is currently the most recent data available on the oral health status of the overall Canadian population, reported that tooth decay affected 57%, 59%, and 96% of Canadian children aged 6-11 years, children aged 12-19 years, and adults, respectively [3]. In addition, the prevalence of early childhood caries (ECC), which is defined as “the presence of one or more decayed (noncavitated or cavitated lesions), missing (due to caries) or filled tooth surfaces in any primary tooth in a preschool-age child, i.e., between birth and 71 months of age” [4] in Canada has been reported to range from 0% to 98.5% with a higher prevalence being found in some communities (eg, indigenous communities and immigrant populations) [5]. Finding ways to decrease the burden of tooth decay is of public health importance.

Tooth decay is a complex and multifactorial condition [6] caused by the metabolism of dietary fermentable carbohydrates (eg, sucrose) in the mouth by bacteria (primarily *Streptococcus mutans*) leading to acid production which can cause tooth structure demineralization [7-9]. Untreated tooth decay in children can interfere with growth, sleeping, eating, and speech development; can cause poor self-esteem; and result in missed school days [4,10-12]. Tooth decay treatment varies, ranging from fluoride application in early stages to more invasive and expensive treatments including fillings, root canals, crowns, and extraction [8]. Tooth decay treatment is also the most common reason for day surgery in preschool-aged children in Canada [13]. Different approaches can be used to prevent tooth decay, including water fluoridation, topical fluorides, oral hygiene (eg, brushing and flossing), pit and fissure sealants, and optimizing dietary intake [14].

More specifically for dietary intake, the foods consumed can both cause and prevent tooth decay [9,15-20], making this relationship complex. For food constituents that cause tooth decay, a diet high in fermentable carbohydrates (eg, sugar) is associated with increased risk [15,21-23]. In addition, frequent consumption of fermentable carbohydrates (eg, sugar) is thought to be associated with more risk [23]. Because of a strong link between sugar and tooth decay, the World Health Organization recommends that intake of free sugars (ie, natural sugars within honey, syrups, fruit juices, and fruit concentrates, as well as simple sugars added to foods or beverages by a manufacturer or consumer) be less than 10% of total energy intake per day; furthermore, they suggest reducing free sugars to less than 5% of total energy due to the cariogenic effects [24]. In addition, the Canadian Pediatric Society recommends that juice should be limited to 125 milliliters per day for children [25]. Although sugar intakes are an important determinant of tooth decay, dietary factors and eating patterns can also have protective properties that minimize tooth decay risk. For example, consuming calcium-rich foods, such as cow’s milk, cheese, and plain yogurt, promotes tooth enamel remineralization, which can assist in tooth decay prevention [26-28]. Furthermore, eating fresh fruit and vegetables, whole grains, and high-quality protein foods is recommended to help minimize tooth decay risk [15]. Consuming xylitol-based products (eg, sugar-free gum) is thought to also assist in prevention of tooth decay due to their ability to increase salivary output, decrease proliferation of cariogenic bacteria, and replace sugary foods in the diet [29,30]. Vitamin D deficiency has also been found to be associated with dental caries [31]. Because of the strong and complex relationship between diet and tooth decay, there is a need to ensure that the public has access to quality information on this topic from various channels.

Written information is commonly used to deliver health information and has been recommended to be used in combination with verbal information to improve patient-related outcomes [32]. This type of information can be delivered using various strategies, including paper-based resources available in clinic settings and online resources (eg, internet and social media). The readability, content, and trustworthiness of written information on various oral health topics have received some attention in the peer-reviewed literature [33-42]. However, studies on written information regarding diet and tooth decay have received less attention. Because providing counseling on diet and tooth decay has been found to be associated with numerous barriers [43], written information may be an important strategy to help disseminate information on this topic; therefore, research in this area is needed. For analyses of written content available on diet and tooth decay, a few previous content analysis studies on paper leaflets have been conducted in the United Kingdom and Australia. These studies found that the nutrition information was at times incomplete, confusing, and contradictory to other nutrition recommendations (eg, national guidelines) [44,45]. Information about written resources from online sources on this topic is limited.

The purpose of this study was to explore information targeted to the public on diet and tooth decay available from websites of professional organizations and regulatory bodies in Canada and the readability of this information. Professional organization and regulatory body resources were chosen as these organizations are known to produce numerous resources on health topics for the public that are easily and freely accessible and are often distributed using numerous channels including online (including social media), dental offices, health units, and schools.

## Methods

This study followed the Standards for Reporting Qualitative Research (SRQR) checklist (Checklist 1) [46].

### Ethical Considerations

This project was determined by the University of Saskatchewan Behavioral Research Ethics Board to meet the requirements for exemption status as per Article 2.2 of the Tri-Council Policy Statement: Ethical Conduct for Research Involving Humans—TCPS 2 (2018) [47]. No research participants were included as part of this study. Informed consent was not needed, because information was available in the public domain.

### Data Collection

From June to August 2020, 2 researchers (MF and JL) thoroughly searched the websites of professional organizations and regulatory bodies for health professionals in Canada for all English language web pages that contained information related to diet and tooth decay targeted toward the public. MF was an undergraduate nutrition student at the time data were collected and analyzed, and JL is an academic dietitian and researcher with research interests in diet and oral health. JL has numerous years of research experience conducting qualitative research and mentored MF throughout the research process. The researchers (MF and JL) first attempted to locate all national and provincial professional association and regulatory body websites for the different health professions (eg, dentists, dental hygienists, dental therapists, dietitians, and physicians) using the Google search engine. Following identification of possible websites, each website was then thoroughly searched by the same 2 individuals for any information on diet and tooth decay; the websites were searched using multiple strategies including searching the terms “diet,” “nutrition,” “food,” “sugar,” “tooth decay,” “dental caries,” and so on, in the website search engine and also exploring website directories for possible web pages containing diet and tooth decay content. We did not limit the search to a specific domain or directory. Because information about diet and tooth decay was found in many places, a series of inclusion and exclusion criteria was developed to determine which web pages were eligible for inclusion.

All web pages from Canadian health professional associations and regulatory bodies with explicit information on diet and tooth decay were included except for curriculum-related materials and association magazines. We included all web pages with diet and tooth decay information (even if diet and tooth decay was not the focus) to ensure that all messaging related to this topic was captured from these websites. In addition, if an oral health professional organization web page had information about the relationship between diet and general oral health (eg, ‘tips for a healthy mouth’ and “good oral health”), these web pages were included. These web pages were included as many of the recommendations relevant to tooth decay were spoken about in broad terms. However, web pages from Canadian non–oral health professional associations and regulatory bodies (eg, dietitian and physician) were included only if they explicitly had content that linked diet to tooth decay. In addition, web pages targeted toward certain oral health conditions (eg, xerostomia, acid reflux, and dental erosion) were included if they explicitly connected the condition to tooth decay and discussed dietary information. Web pages on fluoride were included only if they discussed dietary fluoride sources (eg, fluoridated water and food sources). Finally, video content was included if the videos were made directly by the association or regulatory body themselves and contained information on diet and tooth decay. In addition, during the data collection phase, duplicate resources were found both within websites and between websites (eg, same resource in PDF format and on a web page). Therefore, if a web page provided a PDF containing duplicate information as on the web page, only the web page was selected. However, duplicate resources found across different websites or web pages were included in the dataset. Any discrepancies between the 2 searches (1 by MF and 1 by JL) were discussed and resolved.

Information about the website and web page characteristics was recorded by MF and verified by JL, including the title of the website and web page, name of organization, date of publication or last update, and URL. This information was organized using Microsoft Excel (Microsoft Inc). All included web pages were downloaded as PDF files. MF and JL met frequently through the data collection process to ensure that the search and data extraction processes were as thorough and accurate as possible.

### Data Analysis

#### Readability Assessment

Two web pages that focused mainly on diet, excluding those specific to fluoride, were selected by MF and JL from each website for readability testing; visual elements such as images and graphics were not included as part of the readability assessment. The web pages focusing on fluoride were not chosen for this analysis as the usual content of these web pages was regarding fluoride varnishes and treatments in a dental office and not diet. If the website had only 1 relevant web page that was included in the dataset, then that single web page was included in the readability assessment. The WebFX Readability Test Tool [48] was used to assess the readability of the selected web pages using a variety of measures, including the Flesch-Kincaid Reading Ease (FKRE), Flesch-Kincaid Grade Level (FKGL), Gunning Fog Score (GFS), Simple Measure of Gobbledygook (SMOG) Index, Coleman Liau Index (CLI), and Automated Readability Index (ARI). This readability test tool has been used in other similar studies assessing the readability of written text [49,50].

FKRE scores are based on a 0‐100 scale, in which a high score indicates a lower readability level; a score between 80 and 90 is equated to a grade 6 reading level [51]. FKGL, GFS, SMOG Index, CLI, and ARI scores all estimate the formal education-grade level needed to comprehend a passage of text [51]. The recommended reading level for the public is no higher than grade 6 [51,52]; therefore, a cutoff of 90.0 to 80.0 for the FKRE test and grade 6.0 reading level for the other measures was used to determine whether the web page met readability guidelines. The web pages were uploaded to the online tool through direct input by MF after excluding any text that was not in full-sentence format (eg, titles, headings, and bulleted lists).

#### Qualitative Analysis

The content of the web page messaging was analyzed using conventional qualitative content analysis [53]. Written data (as well as images) were coded inductively (without the use of preconceived categories) and organized into larger categories, subcategories, and themes by MF. NVivo software (version 12, QSR International) was used for data analysis. A second researcher with qualitative research experience (JL) read all included web pages and met numerous times with MF during the data analysis process to discuss code conceptualization and the themes that were being developed. These meetings helped to lead to new insights and better articulation of ideas and were important to promote trustworthiness regarding the findings [54]. Discrepancies between MF and JL were addressed through collaborative online discussions, where both researchers explained their reasoning and revisited the data together until they reached agreement. This approach helped ensure consistency and depth in the analysis.

In addition, as the analysis proceeded, we also recorded the number of web pages that provided information on key topic areas in the web pages including mentions of sugar or sweet or sugary or sugars, and so on, bottle and sippy cup use, avoiding dipping pacifiers in sweet substances to soothe an infant, snacking, Canada’s Food Guide, water, and cheese as a snack. These numbers were determined by MF and verified by JL.

## Results

### Overview

In total, 23 Canadian health professional association or regulatory body websites were found to contain information on diet and tooth decay (or overall oral health as mentioned previously). Website characteristics are listed in Table 1. Most websites were from provincial dentist and dental hygienist organizations.

**Table 1.** Canadian professional association website characteristics that contain information on diet and tooth decay (n=23 websites).

	Values, n (%)
Website organization	
Dentist	12 (52)
Dental hygiene	7 (30)
Medical	2 (9)
Dental therapy	1 (4)
Dietitian	1 (4)
Organization level	
Provincial organization	17 (74)
National organization	6 (26)

Within these 23 websites, 213 web pages were collected, including 188 written resources, 11 videos, and 14 children’s activities (eg, coloring sheets, word searches, and doorknob hangers) that contained diet and tooth decay information. Moving forward, these resources will all be described as web pages.

In total, 38 out of 213 (18%) web pages were focused directly on diet (eg, had diet- or food- or feeding-related words in the title), excluding those specific to fluoride. An additional 16 out of 213 (7.5%) included web pages focused on fluoride. Of note, many web pages targeted parents and school-aged children; fewer web pages targeted other age groups, including adolescents and older adults.

Web pages that focused mainly on diet and tooth decay had titles such as “Healthy Eating for Children,” “Nutrition & Oral Health,” and “Your Child’s Diet.” The remaining web pages with information on diet and tooth decay did not focus on this topic and instead had other focuses with small amounts of information about diet and tooth decay included. Examples of these non–diet-focused web pages were oral hygiene tips for infants and children, general oral health tips, information about specific dental conditions, oral health guides for caregivers of children and seniors, cannabis factsheets, dental hygienist factsheets, frequently asked questions pages, web pages targeted to retirees focused on dental health, and dental insurance resources.

### Readability Analysis

Table 2 outlines readability measures for a sample of 37 diet-specific web pages. In total, only 5 web pages (14%) met the recommended 90.0 to 80.0 range for the FKRE test. Similarly, only 27%, 3%, 22%, and 19% of web pages met the grade 6.0 reading level cut-off for the FKGL, GFS, SMOG, and ARI tests, respectively. The CLI test found that all web pages scored higher than a grade 6.0 reading level.

**Table 2.** Readability test scores for Canadian professional association web pages that contain information on diet and tooth decay (n=37 web pages).

	Overall mean (SD)	Lowest score	Highest score
Flesch Kincaid Reading Ease	70.0 (10.9)	51.4	96.6
Flesch-Kincaid Grade Level	7.1 (1.8)	1.3	10.5
Gunning Fog Score	9.5 (1.9)	4.8	13.3
SMOG Index	6.9 (1.4)	3.9	9.8
Coleman Liau Index	11.5 (1.5)	8.1	13.8
Automated Readability Index	7.5 (2.1)	0.8	11.1

### Content Analysis

#### Overview

Messaging depth varied across all web pages, ranging from general advice (eg, eat a “healthy diet” and “balanced diet”) to specific recommendations (eg, specific number of meals and snacks to consume per day and amount of juice to drink per day). Three major themes were identified: Foods, Beverages, and Behaviors to Limit; Foods, Beverages, and Behaviors to Choose; and Mixed and Other Unclear Messages. These themes are described in detail in the paragraphs below.

#### Foods, Beverages, and Behaviors to Limit

Websites varied in their recommendations of which foods, beverages, and behaviors to limit. While some web pages stated that all foods can cause tooth decay (no matter what or how you eat), many web pages targeted (1) sugar, carbohydrates, and acid; (2) feeding children younger than 2 years; and (3) snacking and frequency of eating. These subthemes are described in more detail in the paragraphs below.

#### Sugar, Carbohydrates, and Acid

Sugar, carbohydrates, and acid were often discussed; however, sugar was the topic most mentioned. In total, 19 out of 23 (83%) websites and 142 out of 213 (67%) web pages mentioned the words “sugar” or “sweet” in any context. Some websites specified the type of sugar they were discussing (eg, natural and added) but most generally stated “sugar.” Sugar was a central topic; therefore, it was embedded within many other themes and will be discussed throughout the “Results” section. The next three paragraphs will describe web page messaging surrounding (1) sugar amounts, (2) the language used to describe sugar and its relationship to tooth decay, and (3) the language used to recommend decreasing sugar consumption.

The overall message surrounding sugar was that it is linked to tooth decay and poor oral health and, therefore, intake amounts should be limited. No website quantified an amount of sugar that may cause tooth decay and poor oral health; however, most did generally state that a diet “high” in sugar or consuming “a lot” of sugar may put an individual at risk. One web page also noted that the amount of sweets eaten is not important; rather, it is the length of time that sweets remain in the mouth that negatively impacts teeth the most.

The language used to describe sugar and its relationship with tooth decay varied. While some web pages generally described sugar as a diet component to limit, others described sugar as “bad,” “very harmful,” and “can cause a lot of damage.” Some web pages also included images to emphasize the latter message (eg, sugar as a cartoon pop can, candy bar, and sugar bug, chasing and attacking a scared cartoon tooth; sugar cubes with unhappy facial expressions). The language used to link sugar and tooth decay and poor oral health also varied in intensity. Some web pages acknowledged that sugar consumption may lead to, contribute to, or promote tooth decay and poor oral health, while others described sugar as being a direct cause. Several web pages described sugar as “feeding” the bacteria in the mouth. “Sugar bugs” were also mentioned in a few web pages directed toward children.

The language used to recommend decreasing sugar consumption varied. While some web pages vaguely stated that sugar was something to “be aware of” or “watch out for,” others said to “limit” or “reduce” sugar with no quantification. One web page recommended that sugar be totally eliminated from the diet. Some web pages discussed avoiding “hidden sugars,” at times listing other names for sugar (eg, maltose) and providing advice on reading nutrition labels to help identify and avoid such sugars. A few web pages also discussed limiting sugar intake as a method for decreasing dental care costs.

Alongside sugar, carbohydrates and acidic foods and beverages were also targeted as harmful dietary components that should be limited. A handful of web pages recommended generally limiting carbohydrates, with 1 web page describing carbohydrates as “decay-causing foods.” Most websites recommended reducing intake of acidic foods and beverages due to their relationship to dental erosion and tooth decay. The language used to describe this relationship mirrored that of sugar, as web pages ranged from stating a general link to stating that acid is a direct cause of dental erosion and tooth decay. The call for limitation was also similar to that of sugar; however, no web pages advised the total elimination of acidic foods and beverages from the diet.

Beyond the general recommendation of limiting sugar, carbohydrate, and acid intake amounts described previously, some web pages targeted which dietary sources to limit. The top 5 sugary foods and beverages to limit across web pages were candy, dried fruit (eg, raisins and fruit leather), juice, soft drinks, and baked goods. Recommendations to limit carbonated beverages (eg, diet and nondiet soft drinks, alcohol, and carbonated water) were discussed in some web pages due to the high acid content; however, sugar was more often targeted than acid. The language used to describe these sugary and acidic foods and beverages varied in intensity across web pages. While some web pages did not use adjectives to describe these foods and beverages, others used adjectives in their descriptions (eg, “cavity-causing,” “decay-causing,” and “bad”). In addition, some web pages identified medicine (eg, cough syrup, and lozenges) as a common source of sugar that should be avoided.

#### Feeding Children Younger Than 2 Years

Foods, beverages, and behaviors to limit in children younger than 2 years was also a common topic. Bottle and sippy cup use was the most common topic and was discussed in 34 out of 213 (16%) web pages. The primary message identified was to limit constant sipping of juice, “milk” (ie, unspecified milk), and formula from a bottle and sippy cup, especially at naptime and bedtime. Some web pages also included breastfeeding limitations, advising that infants should not sleep at the breast. Some of these web pages further explained that sugars within nonwater liquids can cause ECC, especially if the infant has constant exposure through a bottle, sippy cup, or breast. Almost all these web pages recommended eliminating bottle and sippy cup use completely in between feedings.

Other infant feeding behaviors such as teething biscuits, dipping pacifiers in sweet substances, and sharing utensils were discussed. In total, 8 web pages recommended avoiding teething cookies, often describing them as “not a good choice” because they promote tooth decay due to their high sugar content. Similarly, 11 web pages recommended to avoid dipping pacifiers in sweet substances (eg, honey and corn syrup) to soothe an infant. Finally, some web pages recommended against sharing utensils with infants, as tooth decay–causing bacteria can be transmitted from the adult’s mouth to the child.

#### Snacking and Frequency of Eating

Snacking messaging cut across almost all websites, primarily within web pages specific to parents and children, adolescents, and under the topics of stress and cannabis. Web pages largely discussed limiting sugary and starchy snacks, as well as limiting the frequency of snacking.

In total, 38 web pages shared a common message of limiting snacking; however, such messaging varied. Some web pages remained general in their recommendation, advising readers to “limit snacking.” Other web pages were more specific by recommending the limitation of sticky snacks and snacks high in sugar, carbohydrates, and acid. Many web pages directly targeted which snacks to limit between meals, most often targeting sticky and sweet foods (eg, candy, fruit leather, chips, and granola bars), carbonated and sugary beverages (eg, soft drinks, energy drinks, and juice), and sometimes fruit (eg, citrus fruits).

Snacking frequency was also commonly discussed; many web pages advised against “constant snacking,” “grazing,” and “frequent nibbling or sipping.” Some of these web pages further explained that frequent snacking, as well as consuming snacks that stay in the mouth for a long time (eg, hard candy and breath mints), increases tooth exposure to sugar and acid. They explained that this exposure can increase the number and length of “acid attacks” within the mouth which can lead to tooth decay. Some web pages also noted that all foods may cause these acid attacks, including nutritious foods, and not just sugary snacks. Multiple web pages stated that “every time you eat” or “each time you eat or drink,” acid attacks the teeth for at least 20 minutes. While some web pages described this process with a neutral tone, others used stronger language. For example, one web page stated that sugar will do more “damage” the longer and more often that the teeth are exposed.

#### Foods, Beverages, and Behaviors to Choose

All websites provided positive suggestions for which foods, beverages, and behaviors to choose to prevent tooth decay and promote general oral health. The main topics discussed across web pages were suggestions to eat a healthy diet, including specific foods, beverages, and micronutrients to choose; healthy snacking; and tooth-friendly eating and drinking. These subthemes will be described in detail in the paragraphs below.

#### Healthy Diet

Many web pages recommended enjoying a “well-balanced,” “healthy,” and “nutritious” diet or to eat foods that are “good for teeth,” without further explanation as to what this recommendation would constitute. However, often these web pages referred the public to another web page within their website for more information on diet. In addition, 19 web pages recommended following Canada’s Food Guide when eating, and some provided active links to the food guide itself, UnlockFood.ca (nutrition information for the public from Dietitians of Canada), and Dietitians of Canada. Of these web pages, one also included the Eat Well Plate as a visual aid. A few web pages stated that the public can consult with their oral health professional for nutritional counseling should more information be needed. Dietitians were referenced within 3 web pages as a profession that dental hygienists work with collaboratively.

When more information about a healthy diet was included, the main message across websites overall was that a healthy diet should contain a variety of foods from the different food groups to minimize tooth decay risk. Not all web pages outlined all 4 food groups (ie, vegetables and fruit, grain products, milk and alternatives, and meat and alternatives), as some left out certain groups, and the names of the food groups varied. However, most web pages explained what the food groups were (eg, dairy, fruits and vegetables, meat and alternatives, and whole grains), and sometimes they provided examples of foods within each group. Multiple web pages also recommended that 3 meals be consumed each day.

#### Specific Foods, Beverages, and Micronutrients to Choose

Although recommendations for a general healthy diet were often broad, web pages also commonly identified specific foods to choose and often discussed the importance of timing of these foods. Cheese was a food that was often highlighted. Cheese was commonly recommended as a healthy food to eat following a meal. Some web pages explained this recommendation, stating that cheese can play a protective role in cavity prevention as it can stimulate saliva flow and neutralize acids within the mouth. Multiple web pages also targeted apples and “juicy fruits and vegetables” as foods to eat after consuming sugary and sticky foods, if tooth brushing is not an option; these web pages sometimes stated that these foods can “clean your teeth.” Finally, the most targeted beverage to choose when thirsty, excluding water (which is described later in this section), was cow’s milk. Some web pages specified that milk should be unsweetened or low-fat.

Websites often discussed the importance of consuming specific micronutrients for optimal oral health. Fluoride was commonly mentioned. Almost all websites identified the benefits of fluoridated water in preventing tooth decay and advised the public to drink fluoridated water if possible. Some websites also mentioned that fluoride can be found in various foods; however, only 1 website provided specific examples (eg, ocean seafood and gelatin). Aside from fluoride, calcium was the most recommended micronutrient. Of the web pages that recommended calcium, only some provided examples of calcium-rich foods (eg, cheese, yogurt, and milk). Most web pages explained their recommendation for calcium, stating that calcium helps “rebuild your teeth,” “make teeth hard,” or “strengthen teeth.” Finally, a small number of web pages recommended a multivitamin for individuals with allergies or restrictive diets (eg, gluten-free, vegetarian, and vegan) to ensure adequate nutrient intake, as they stated that vitamin deficiencies may cause increased risk of tooth decay. A couple of web pages also highlighted other micronutrients as being beneficial for oral health (eg, vitamin D, phosphorus, vitamin A, and vitamin C). In addition, a few web pages mentioned that anemia, iron deficiency, and vitamin D deficiency are more likely to occur in children with severe ECC.

#### Water

In total, 19 out of 23 (83%) websites specifically identified water as being the main beverage to choose in any context. Some web pages described water as providing “sugar-free hydration,” and 1 web page stated that all foods and beverages can contribute to tooth decay except water. Web pages varied in their recommendations, ranging from general (eg, “drink water“ and “choose water”) to specific (eg, amount of water per day). Many web pages stated that water should be chosen to quench thirst, to have between and with meals, and to be kept readily available (eg, have a pitcher of water on the table or in the fridge, carry a reusable water bottle, and keep a glass of water handy at the bedside). Most web pages also suggested choosing water to replace sugar-containing beverages (eg, offer water instead of juice). Some web pages also recommended carbonated or sparkling water as a healthy alternative to other beverages.

Specific circumstances in which water would be beneficial to minimize the risk of tooth decay were also identified across web pages. First, water was recommended by many web pages to be used as a rinse after eating, drinking alcohol, eating sugary foods and beverages, or taking medicine sweetened with sugar. Some of these web pages further explained that water can help “rinse” or “wash” away any sugars and acids that may lead to tooth decay. Second, several web pages recommended that individuals experiencing dry mouth should drink or sip on water to avoid tooth decay. Third, many web pages stated that water is the only appropriate content for bottles and sippy cups, should a bottle or sippy cup be offered outside of mealtimes, as constant exposure to other beverages may cause tooth decay. Some web pages also recommended that non–water bottle contents (eg, milk and juice) be watered down gradually until only water remains.

#### Xylitol and Sugarless Gum, Mints, and Candy

Similar to using water as a rinse, xylitol and sugarless gum, mints, and candy were often recommended for tooth decay prevention. While some web pages advised that such items be chewed or sucked on immediately after eating, especially after eating sticky and sugary foods, others specified that individuals experiencing dry mouth would benefit as well. A few of these web pages further explained that xylitol and sugarless gum, mints, and candy stimulate saliva flow and help “wash away” sugar from teeth. Furthermore, some web pages stated that xylitol can neutralize the acids in the mouth that are produced after eating. One web page described xylitol as “one of nature’s most powerful weapons against dental caries.”

Of the web pages that recommended xylitol, 2 web pages discussed the amount of xylitol that should be consumed to effectively prevent tooth decay. One web page recommended consuming 5‐10 grams of xylitol per day with no mention of frequency, while the other recommended 4‐10 grams of xylitol 3‐7 times a day for 3‐7 minutes at a time. The latter web page noted that frequency is more important than quantity.

#### Healthy Snacking

Healthy snacking was a common topic among almost all websites. A variety of adjectives were used to describe healthy snacking including “healthy,” “mouth-healthy,” “dentally healthy,” “safe,” “tooth-friendly,” and “smart” snacks. Healthy snacking messaging ranged across web pages from offering general recommendations (eg, “choose healthy snacks”) to providing tangible examples. Most web pages provided a healthy snack suggestion as an alternative to a specific food to avoid (eg, snack on an apple instead of candy). The top 5 most mentioned snacks to choose for tooth decay prevention were cheese, nuts, apples, raw vegetables, and plain yogurt. Popcorn was also mentioned across multiple websites as a healthy snack. In total, 14 out of 23 (61%) websites and 36 out of 213 (17%) web pages recommended cheese as a snack. This recommendation was identified in numerous web page forms, including coloring sheets, healthy school snack and lunch ideas, and general “healthy snacking” lists. Every web page that outlined a healthy school snack and lunch recommended cheese (eg, “cheese,” “cheddar cheese cubes,” “cottage cheese,” and “shredded Monterey Jack cheese”), with some web pages mentioning cheese numerous times. In addition, when apples were recommended as a snack, many web pages provided an image of an apple to coincide with the text (eg, cartoon apple and child eating an apple).

In addition to healthy snack ideas, some web pages also discussed healthy snacking behaviors to choose to minimize tooth decay risk. Recommendations varied in topic and depth, as some focused on making healthy snacks more available (eg, stock the pantry and fridge, keep healthy snacks on low shelves for children, and pack healthy snacks if leaving home) while others focused on the timing of snacks (eg, set specific snack times to avoid grazing, set a treat time, and schedule snacks in the midmorning and midafternoon). Numerous web pages quantified the number of healthy snacks to be offered per day, ranging from 1 to 3 times per day. However, other web pages were more general, such as one web page that generally advised “regular” snacks without further explanation as to what “regular” entailed. Some web pages also recommended that healthy snacks be chosen from at least 2 food groups, and 1 web page provided images of these snack combinations (eg, veggies and cheese and fruit and milk).

#### Tooth-Friendly Eating and Drinking

Rather than recommending the total elimination of cariogenic foods and beverages, many websites provided healthy suggestions for eating and drinking sugary foods and beverages to minimize tooth decay risk. If sugary foods or beverages were to be consumed, many web pages suggested enjoying them at mealtimes rather than between meals. Some of these web pages further explained that saliva levels are higher during mealtimes; therefore, sugar could be more easily washed away. Some web pages also mentioned that chocolate is a good alternative to sticky foods, such as chips and candy, because chocolate is easier to rinse away from teeth. In addition, a few web pages recommended eating carbohydrates as a part of a meal; however, no further explanation was provided.

Some web pages also identified fruit as a food to eat with a meal or as a dessert due to its acidity and sugar content. One web page recommended eating fruit over a small time if eaten as a snack (eg, have 10 strawberries or grapes at once rather than nibbling on one every few minutes). A handful of web pages also suggested choosing a piece of fruit over juice. If juice was to be consumed, web pages often quantified a daily maximum amount that varied across web pages, ranging from vague (eg, one serving per day) to specific amounts (125 mL [4 oz], half cup per day). When talking about juice, many web pages advised that juice be offered only as a part of a meal, not as a snack. Finally, 2 web pages recommended using a straw if consuming a sugary beverage and to drink them quickly to minimize contact with teeth.

#### Mixed and Other Unclear Messages

Minimal mixed messaging between and within web pages was found. In a few instances, some foods and beverages were subject to controversy, including recommendations of fruit as a snack between meals, dried fruit, milk in infant bottles, and carbonated or sparkling water. For each of these food and beverage items, there were mixed messages to consume and limit consumption. As mentioned previously, juice and dried fruit were also recommended as a snack in single instances, which is another mixed message. In addition, one web page recommended that sugar be totally eliminated from the diet; however, after providing tips for how to eliminate sugar, it stated in closing that sugar can be part of a healthy diet in moderation. Despite these few mixed messages, there was no widespread debate on these topics across web pages, as the majority shared consistent messaging.

In a few cases, some web page messaging was unclear. For example, sometimes foods were highlighted as healthy options to choose, but it was not clear as to what they actually were (eg, gelatin). Finally, as discussed previously, websites often did not quantify the amount of foods or beverages to consume (eg, “limit” sugar, eat sugar in “moderation,” and “eat cheese”); therefore, it was unclear as to how much of a food or beverage was recommended.

## Discussion

### Principal Findings

To our knowledge, this is the first study that has examined consumer-based resources (primarily written) on diet and tooth decay from websites of professional organizations in Canada. Overall, we felt that there was an impressive number of web pages from these organizations that contained information on diet and tooth decay (213 web pages on 23 Canadian websites). Diet and tooth decay were also covered on web pages that focused on various topics, some of which were not expected to contain this information. The high frequency of this information suggests that professional organizations recognize a strong and important link between diet and tooth decay and general oral health in many different oral health contexts and are passionate about disseminating this message. This work also provides an inventory of how diet and tooth decay messaging is provided in written resources and may be useful for the development of resources on this topic in the future.

Overall, the diet and tooth decay messaging found on the web pages analyzed as part of this study aligns with studies conducted in other countries that have examined the content of diet and tooth decay information available to the general public. For example, messaging surrounding sugar was very common on the web pages analyzed as part of this study (ie, 67% of web pages mentioned “sugar” or “sweet” in any context) as well as other studies that have examined nutrition and oral health messaging in paper-based leaflets. For example, a study conducted in Australia by Arora et al [45] found that 20 of 43 leaflets recommended decreasing consumption of sugary and/or acidic foods and 23 of 43 leaflets recommended decreasing the consumption of sugary and/or acidic drinks. As well, a study conducted in the United Kingdom by Morgan et al [44] found that messaging surrounding sugar was also common; for example, they found that 27 of 30 leaflets recommended limiting consumption or reducing the frequency of sugary foods. As well, Long et al [55] found that 31 of 42 YouTube videos on nutrition and dental caries contained some type of message on sugar. Messaging surrounding water was also common in this study (19/23, 83% of websites) as well as the studies by Arora et al [45] and Morgan et al [44]. More specifically, Arora et al [45] found that 35 of 43 leaflets recommended water, and Morgan et al [44] found that 21 of 30 leaflets mentioned to drink only milk and water. However, Long et al [55] found that only 12 out of 42 YouTube videos on diet and tooth decay contained messaging on water. Finally, cheese as a snack was also a common recommendation in this study (61% of websites), which aligns with the studies also conducted by Arora et al [45] and Morgan et al [44]. Morgan et al [44] and Arora et al [45] found that 16 of 30 and 16 of 43 leaflets recommended cheese as a snack, respectively.

Although the large number of web pages available from Canadian health professional associations with information on diet and tooth decay is positive as it emphasizes the importance of this topic, the usefulness of this information may be limited in some cases. Web pages on topics separate from diet oftentimes included single, general recommendations related to diet (eg, “limit sugar” and “eat a healthy diet”). In this study, these statements commonly appeared on web pages with short lists of various oral health tips (eg, tooth brushing and flossing and tips for a healthy mouth). Similar results were found in related studies of nutrition and oral health resources performed in the United Kingdom and Australia as mentioned previously. These resources were oftentimes vague surrounding the amount, timing, and type of sugary foods and beverages to limit, and the Australian resources often did not explain what constitutes a healthy diet [44,45]. Although such information does link diet to tooth decay and overall oral health, the generality of such messaging may have narrow usefulness to the public and may limit their ability to follow recommendations.

More specifically, messages to “limit sugar” or to “limit sweets,” without providing further information, assume that the public would know which sugary foods and beverages to limit and the amount in which those foods should be consumed. However, this assumption may not be true for everyone. Previous research has found a lack of knowledge among the public regarding sugar, including sugary foods and beverages [56-59]. In addition, although limiting sugar was discussed across almost every website, no website quantified a maximum daily amount recommended. The general language used by some web pages was difficult to quantify (eg, avoid having a diet “high” in sugar or “a lot” of sugar). Therefore, if referring to limitation, it may be effective for resources to provide the public with a recommended target maximum amount for daily sugar consumption. For example, a recommendation would be to consider using the World Health Organization’s recommendation for free sugar intake (less than 10% of energy intake, with additional benefit of lowering to 5%, of total energy intake to prevent tooth decay) [24]. This quantification could be translated into practical terms for the public to understand (eg, 5% of total energy intake equals approximately 6 teaspoons of sugar [24]), which could be used in correspondence with visual aids which have been found to be helpful for nutrition education [60]. It may also be effective to demonstrate how many teaspoons of sugar are within specific food or beverage items (eg, soft drinks and candy).

In addition, the general recommendation to eat a “healthy,” “well-balanced,” and “nutritious” diet, with no follow-up information, assumes that the public would already understand what would constitute such a diet. This assumption was evident as oftentimes no further information (eg, food or beverage examples) coincided with this recommendation. Prior research has demonstrated that although the public oftentimes has a broad understanding of healthy eating and can acknowledge its importance, the application of such nutrition knowledge within daily life is difficult [61,62]. Numerous factors influence the discrepancy between knowledge and action in the context of healthy eating, one of which may include a lack of procedural knowledge (“knowing-how”) in addition to declarative knowledge (“knowing-that”) [62]. For example, while it is important to know that frequent and prolonged consumption of sugary beverages, such as soft drinks or fruit juice, may promote tooth decay (declarative), it is equally important to know that water is a healthy alternative to choose instead (procedural). Some web pages linked such messages; however, not all did. Therefore, a strategy for future resource development is to provide tangible examples of foods and beverages to choose so that the public can use their procedural knowledge. These food or beverage examples could be framed as alternatives to cariogenic foods or foods to pair with cariogenic foods.

Providing general healthy eating recommendations also assumes that the public would know and be able to find credible external resources to refer to for additional information on this topic. Some examples of credible resources include registered dietitians, other health professionals, regional health authorities, and federal and provincial government resources (eg, Public Health Agency of Canada and Canada’s Food Guide). Although 19 web pages referred the public to Canada’s Food Guide (and sometimes provided active links), prior research has demonstrated that Canadians largely have a relatively low reported use and low reported knowledge of Canada’s Food Guide despite relatively high awareness of this document [63,64]. Therefore, despite the direct link, Canadians may still face the challenge of navigating and using this resource. Since most web pages did not provide direct referrals, it may be even more unlikely that the public would be able to refer to such resources due to the lack of knowledge suggested through previous research. Providing direct information about where the public can go for information is important as the use of potentially noncredible nutrition information is common.

Interprofessional collaboration is important as different professions have unique and specialized perspectives to help optimize the patient care and the health of populations. In this study, a need for improved collaboration between dental and dietetic professionals was identified. This collaboration could take on many forms, including referrals between the different providers and collaborating to develop resources and presentations for professionals and the public. Although 3 oral health web pages referenced dietitians as health care professionals that work collaboratively with dental hygienists, very few web pages referred the public to dietitians for assistance regarding eating to prevent tooth decay. Prior research and position statements have suggested that improved dietetic and dental collaboration is needed and can help improve oral health promotion, client-centered care, and disease prevention [15,65-67]. Depending on the patient, dental recommendations regarding eating could also differ from dietetic recommendations due to differences in professional perspectives; these differences have been reported previously [65,68,69]. For example, some websites in this study recommended eating no more than 3 meals and 2-3 snacks per day with only water in between meals. While this recommendation may be in congruence with the recommendation to minimize teeth exposure to foods and beverages other than water, this may oppose the common recommendation to eat intuitively. Intuitive Eating is a flexible style of eating that encourages an individual to intuitively gauge when to eat, what to eat, and when to stop eating largely through recognizing and following internal sensations of hunger and fullness [70]. As a result, the dental priority of minimizing teeth and food interaction may be incongruent with common dietetic recommendations of following a more flexible eating pattern. Therefore, it is important that all health professionals are familiar with the types of recommendations of their interprofessional colleagues and collaborate with one another to clarify and improve messaging to decrease confusion and promote better outcomes. More training on diet and oral health in health professional training programs is also a strategy that could be used to help address this issue [15,71].

Although mixed and unclear messaging was minimal across web pages, it was still present. Lack of clarity surrounding certain foods to choose and limit may form areas of potential confusion for the public. For example, it may be challenging for a parent to know whether dried fruit is a tooth-friendly snack for their child when one resource recommends limiting its consumption while another identifies it as a healthy snack. Furthermore, it may be challenging for the public to seek out recommended foods when it is not clear as to what these foods are (eg, gelatin). Therefore, as health care professionals and resource developers, it is important to clarify as much as possible what one’s recommendations are as they could easily be misinterpreted by the public.

After analyzing the readability of the tested sample of web pages, we found that many web pages scored higher than a recommended grade 6 reading level. This finding aligns with other studies that also analyzed the readability of various online resources targeted toward the public, including those released by associations [34,35,72-75]. Providing written information that is accessible to individuals with lower literacy better allows for improved reading comprehension, thereby helping minimize health disparities, such as tooth decay.

### Limitations

One limitation of this study was that the data were collected in 2020. However, most of the websites reviewed in the original study are still active and contain similar content; only some websites are no longer available or have removed the relevant resources. Among the active websites, many web pages analyzed in 2020 are still accessible at the same links with similar content. As well, in many cases, although the content analyzed in 2020 remained largely the same, it may have been moved to new locations due to website redesigns. Overall, we found that the content remained relatively consistent over time, suggesting that the findings from the original analysis would be similar should the data collection process be repeated today. In addition, no major changes in recommendations and evidence related to diet and tooth decay have emerged since these data were collected that would substantially change these messages. This study also serves as a valuable reference point for tracking changes over time. Another limitation of this study was that only a select sample of web pages was tested for readability due to the larger than expected dataset. A possibility exists that these selected web pages may not provide an accurate readability evaluation of the website’s entire content on diet and tooth decay. In addition, the WebFX tool may not provide a perfect assessment of readability. We also did not contact the organizations to ensure that we had all their public web pages on diet and tooth decay. As well, since the information was present across the websites in numerous different types of places, it may be possible that not all web pages were found. However, 2 researchers independently and thoroughly searched the websites to ensure that all content was captured. We also included only English language web pages in this study, which could result in missing critical information that caters to non-English-speaking populations in Canada. In addition, we did not analyze social media content provided by these organizations which are also important means of conveying health information. By not including these elements, our assessment may be incomplete. As well, we did not examine differences in messaging between different types of organizations, which is an area for future research. Finally, because our study focuses on websites of professional organizations and regulatory bodies in Canada, it is not representative of the entire online landscape of diet and tooth decay resources available to Canadians. Future research could assess the content of other types of resources available on diet and tooth decay from different sources and organizations.

### Conclusions

Overall, an abundance of information is available to the Canadian public on diet and tooth decay from professional associations and regulatory bodies. The dominant message across web pages was to limit sugar and to eat a healthy diet, although the explanation of this message varied in depth between web pages; as well, most of the information was targeted toward children and their parents. Of note, this information was found in many different forms under a wide variety of web page topics throughout the websites. Mixed messaging was present (eg, surrounding dried fruit) but was limited. In addition, the readability of web pages often exceeded the recommended grade 6 level, which is something to consider when creating future content on this topic. The development of national guidelines related to diet and tooth decay prevention would be helpful to ensure that consistent and clear messaging is available on this topic for health professionals and the public; this process will require extensive collaboration between experts from a variety of disciplines to ensure their applicability to the Canadian population. In addition, future research is needed to understand the perspectives of the public on resources for diet and tooth decay and how individuals interpret different types of messaging on this topic. Intervention studies could also be conducted to determine the impact of different types of messaging on knowledge, attitudes, practices, and oral health outcomes. These activities will all help to decrease the burden of tooth decay in Canada and beyond.

## Supplementary material

10.2196/59474 Checklist 1 SRQR (Standards for Reporting Qualitative Research) checklist.

## Abbreviations

ARI: Automated Readability Index
CLI: Coleman-Liau Index
ECC: early childhood caries
FKGL: Flesch-Kincaid grade level
FKRE: Flesch-Kincaid Reading Ease
GFS: Gunning Fog Score
SMOG: Simple Measure of Gobbledygook
SRQR: Standards for Reporting Qualitative Research
